# Occupational violence and burnout among teachers: a narrative review

**DOI:** 10.47626/1679-4435-2022-602

**Published:** 2023-02-03

**Authors:** Beatriz Maria dos Santos Santiago Ribeiro, Júlia Trevisan Martins, Bruna Aparecida dos Santos Santiago Ribeiro

**Affiliations:** 1 Enfermagem, Universidade de São Paulo, Ribeirão Preto, SP, Brazil; 2 Enfermagem, Universidade Estadual de Londrina, Londrina, PR, Brazil; 3 Educação, Escola Estadual Hatsue Toyota, Marília, SP, Brazil

**Keywords:** occupational violence, teachers, burnout syndrome., violência no trabalho, professores escolares, esgotamento profissional.

## Abstract

Occupational violence may favor the development of burnout syndrome. The objective of this study was to identify which characteristics are associated with burnout syndrome in teachers exposed to occupational violence and discuss measures aimed at reducing this type of violence. A narrative review with a theoretical-reflective approach was conducted on SciELO library and on PubMed, Web of Science, and Scopus databases. The violence experienced by teachers causes health-related problems and illnesses, especially in mental health, favoring the development of burnout syndrome. Occupational violence has affected teachers and influenced the onset of burnout syndrome. Thus, plans and actions involving teachers, students and their parents/legal guardians, employees, and especially managers are essential to promote safe and healthy work environments.

## Abstract (pt)

A violência no trabalho pode favorecer o surgimento da síndrome de *burnout*. Objetivou-se conhecer as características dos professores vítimas de violência ocupacional que podem ocasionar a síndrome de *burnout* e refletir sobre as medidas utilizadas para a redução dessa violência. Realizou-se uma revisão narrativa, juntamente de uma reflexão teórica-reflexiva. Foram conduzidas buscas através da biblioteca SciELO e das bases de dados PubMed, *Web of Science* e Scopus. A violência sofrida pelos professores provoca danos e agravos à saúde, em especial a saúde mental, e, por sua vez, favorece o surgimento da síndrome de *burnout*. Afirma-se que a violência tem acometido os professores e favorecido o desencadeamento da síndrome de *burnout*. Assim, são necessários planejamentos e ações que envolvam os professores, os alunos e seus pais ou responsáveis, os funcionários e, sobretudo, os gestores, a fim de propiciar um ambiente laboral seguro e saudável.

## INTRODUCTION

The main purpose of the school environment is to promote the development and growth of both students and teachers, but it has been the target of increasing violence.^^1^^ School violence is a complex phenomenon and, to successfully combat it, everyone involved in the teaching-learning process must participate. Family members, teachers, students, schools, and government agencies should discuss this topic and jointly seek effective ways to combat school violence. It should be noted that there are several factors responsible for school violence.^^2^^

To understand the school context, one should know that teachers perform numerous functions within the educational setting, including the teaching of children, adolescents, young adults, adults, and older people. Thus, teachers play a fundamental role in the educational process, given that they are the ones to introduce cultural aspects to children and reinforce teachings related to ethical, moral, and aesthetics values, as well as school subjects.

Manifestations of violence in the school environment may be symbolic, subjective, or objective.^^3^^ Subjective or open violence is the most perceived type of violence because it involves an exceeding amount of strength and the aggressor can be easily identified.^^4^,^5^^ Symbolic violence is perceived as invisible because it is characterized by language, words, and nominations, making its recognition neglected in most cases. Importantly, this type of violence promotes discrimination and social exclusion, in addition to having destructive effects on those who experience it.^^4^^ Finally, objective or systemic violence is related to exploitation in social, economic, or political relationships and may also be perceived as invisible, considering that this type of violence is often considered inherent to social relationships.^^5^^

The phenomenon of violence may assume different compositions and social practices, varying according to time and place and occurring implicitly or explicitly. Implicit violence is considered a precursor of other types of violence. Conversely, explicit violence is easily recognized as such and perpetuates the delusional belief that it is the only harmful type of violence.^^6^^ Examples of implicit violence include illiteracy, low wages, corruption, unemployment, hunger, impunity, and prejudice, whereas examples of explicit violence include robbery, homicides, sexual assault, and kidnapping.

Occupational violence can be both physical and verbal and may lead workers to manifest psychosocial changes, as well as decreased work performance. In fact, the aggressor may sometimes be another company employee.^^7^^ Teachers are exposed to psychological violence mostly through verbal aggression and threats,^^8^^ and this issue occurs in several parts of the world.^^9^,^10^^ Thus, occupational violence is a phenomenon that is related to other types of social violence.^^11^^

Occupational violence may lead teachers to feel stressed, scared, and insecure, thus favoring the onset of burnout syndrome (BS). BS is described as a prolonged response to chronic work-related emotional and interpersonal stress. It results from chronic stress related to occupational activities, causing issues at the personal, professional, family, and social levels.^^12^^

BS is manifested through four main symptoms: physical, psychological, behavioral, and defensive. Physical symptoms include constant fatigue, sleep disturbances, lack of appetite, and muscle pain, whereas psychological symptoms include lack of attention, memory problems, anxiety, and frustration. Behavioral symptoms are characterized by negligence at work, irritability, inability to concentrate, increased conflicts with colleagues, and long breaks. Finally, defensive symptoms are those related to isolation, feelings of inferiority, and decreased work quality.^^13^^

In view of the aforementioned, studies focused on occupational violence among teachers are relevant because they can assist teachers, managers, and society in seeking strategies to minimize this type of violence and, consequently, maximize the quality of life of those involved. The objective of this study was to identify which characteristics are associated with BS in teachers exposed to occupational violence and discuss measures aimed at reducing this type of violence.

## METHODS

A narrative review with a theoretical-reflective approach was conducted on SciELO library and on PubMed, Web of Science, and Scopus databases using the following keywords: violence, burnout syndrome, occupational burnout, teachers, strategies, and work. Additional theoretical backgrounds on the subject were used, as well as the Occupational Health discipline from the Postgraduate Program in Nursing at the State University of Paraná. Studies that were in accordance with the objectives of the present study were selected, with no restrictions on date of publication.

## RESULTS AND DISCUSSION

### IDENTIFYING CHARACTERISTICS THAT MAY TRIGGER BS IN TEACHERS EXPOSED TO OCCUPATIONAL VIOLENCE

The authors who analyzed the database of the Registry of School Occurrence/Secretariat of Education of the State of São Paulo (ROE/SEE-SP) reported that age is a factor for increased rick of violence in teachers.^^14^^ Studies show that younger teachers are more likely to suffer violence in the workplace.^^10^,^15^^ Older teachers have more teaching experience and, therefore, can deal with conflicts more easily, showing greater resilience and lower levels of work-related stress.^^16^^

Furthermore, age indirectly impacts physical violence and is also associated with working conditions, as evidenced by temporary contracts, which are more common among younger workers.^^17^^ Temporary teachers have worse working conditions than teachers with a stable employment relationship. When entering the work environment, teachers may be disappointed with the real working conditions and may become dissatisfied with their job.^^18^^

Another factor that may influence violence is time of experience, which has been shown to assist in the control of aggressions and threats.^^14^^ According to Gerberich et al.,^^19^^ teachers with more professional experience had lower rates of violence. The authors also highlight that teachers who work full-time in the United States (USA) suffer violence in greater proportions, which may be related to increased exposure time and greater dedication to students. Conversely, Wei et al.^^15^^ found that substitute or part-time teachers had higher rates of violence.

The public education network in the state of São Paulo offers incentive pay to teachers who work in violent communities. According to their time of experience, teachers have the priority to choose which school they will work at annually, meaning that more experienced teachers can choose to work at schools with higher salaries, but which are also more vulnerable and violent.^^14^^ Higher qualifications are not always combined with a greater ability to control student behavior. However, increased experience often helps to control aggressions and threats.^^14^^ Teachers with higher degrees are more likely to be victimized when compared with teachers with a bachelor’s/teaching degree.^^15^^ Of note, university professors also suffer some types of violence.^^20^^

Teachers who only attended teaching training courses have more skills to deal with issues of violence than teachers with higher education or graduate degrees. This issue may be associated with the fact that teacher training courses insert professionals earlier in the labor market, increasing their experience. However, there are no statistically significant relationships between teacher qualifications and other types of violence.^^14^^

Studies have investigated the variables gender, race/skin color, sexual orientation, and religion or beliefs to identify factors that may increasingly expose teachers to violence. Repeated exposure to violent acts causes suffering and negatively impacts health.^^21^-^23^^ The 2011 Education at a Glance report points out that elementary and high school classes in Brazil have approximately 30 students, although they should have a maximum of 24, thus exceeding the median calculation. However, class size varies between public and private schools. Although private schools have a considerable number of students per class, public schools have seven more students per teacher compared with private schools.^^24^^

The Teaching and Learning International Survey (TALIS) reported that Brazilian teachers teach an average of 25 hours per week, 6 hours more than the average of other countries, in addition to spending 10-22% more time working on activities such as homework and orientation.^^25^^ Regarding the number of students, small (less than 10 students) or large (more than 25 students) classes can influence the occurrence of violence.^^15^^ Teachers cited class size as a factor associated with violence. As for punishments, they must be effective and protect both teachers and aggressors, given that the lack of guidance/counseling increases violence.

Still, it is common for students to hide markers and chalk in an attempt to delay the beginning of the class or to prevent it from beginning at all. They throw chalk at teachers and boo at them with the insistent goal of disrupting the class. There have been cases of serious burns caused by the presence of corrosive glue on the teacher’s chair, petitions without the slightest justification by groups of students, and anonymous complaints by students based on untrue facts with the goal of disrupting work performance,^^26^^ among other issues.

Poor working conditions can make teachers more vulnerable to physical violence, meaning that schools located in vulnerable regions are more likely to experience violence.^^17^^ McMahon et al.^^27^^ found higher rates of violence against teachers in urban areas than in rural and suburban areas in the USA. However, the present study did not include spatial data.

Regarding race/skin color, there were no statistically significant associations. Contradictory to McMahon et al.,^^27^^ African-American teachers reported less rates of violence.^^27^^ Teachers without a partner had higher rates of violence, given that a partner may mitigate the effects of violence by providing support.^^15^^ Other factors that may expose teachers to greater risks of violence are sexual orientation and religion or beliefs.^^17^^ Maternal education was also shown to impact the rates of violence: the higher the maternal education, the lower the chances of conflicts with colleagues.^^14^^ Importantly, teachers are being affected by BS, and this profession is still mostly composed of women.^^28^^

Prolonged exposure to negative occupational factors over time leads to continuous states of concern, which may exceed emotional limits. This leads to work-related feelings of cynicism, impotence, and emotional exhaustion, which in turn contribute to decreased vitality and dedication, thus resulting in dissatisfaction, desire to change jobs, and absenteeism.^^29^^ Violence obviously promotes negative feelings in teachers, contributing to a lack of motivation and affecting their performance in the teaching-learning process.^^1^,^2^^

A study investigating BS, ethical leadership, and coping strategies among teachers in the Federal District reported that BS may lead to absenteeism and presenteeism, and thus multidisciplinary knowledge on the subject should be further expanded to develop coping strategies for issues that harm both the physical and mental health of teachers.^^30^^ However, we believe the aforementioned studies may help to identify characteristics that may expose teachers to increased risk of occupational violence and BS in the future.

### MEASURES TO REDUCE OCCUPATIONAL VIOLENCE IN THE SCHOOL SETTING

Preventing occupational violence is imperative, considering that it contributes to work accidents, absenteeism, and suffering, which may cause mental health problems.^^31^^ Measures aimed at preventing violence should be holistic, interdisciplinary, and permanent and should encourage democratic relationships, the harmonious coexistence of all school members, and respect for differences. Public policies are also fundamental, given that violence has varied causes.^^32^,^33^^

A literature review on ways to prevent school violence described actions aimed at improving the organizational climate and promoting a culture of peace at schools, such as opening schools on weekends and conducting school activities focused on conflict resolution. These actions should be conducted during individual interventions with students, professional guiding sessions, and actions promoting coexistence rules; they should be permanent, systematic, and consider the singularities and specificities of each school.^^32^^

The quality of the interaction between students and teachers positively impacts the prevention, reduction, and resolution of violent episodes. Nurturing close relationships in school makes this environment more welcoming and human, thus reducing the risk of violent actions.^^14^^ According to Tavares & Pietrobom,^^14^^ parental participation in school activities has a significant association with lower levels of school violence, that is, students with a structured family nucleus are less likely to engage in violent behavior.

Regarding public policies, the Program for the Prevention of Violence Against Teachers (Programa de Prevenção à Violência Contra Educadores, PNAVE) was created on December 11, 2013 with the goal of developing punitive measures for students or employees who commit violent acts. The main proposals mentioned in the document suggest the implementation of educational campaigns, whose objectives include preventing physical and moral violence without embarrassing teachers; permanently or temporarily suspending aggressors (students or employees), according to the severity of the offense; transferring students who commit violent acts to another school, after analysis by the educational authorities; granting temporary leave to teachers who are at risk, without losing their salaries; and fining or imprisoning aggressors from 3 to 9 months.^^34^^ Although PNAVE was created, few changes have been made to the school environment to prevent acts of violence against teachers, employees, and students.

A study^^35^^ showed that strategies used by teachers to combat school violence include lectures; meetings with the family; dissemination of legislations and the school’s internal regulations; meetings with teachers and the school council, the secretary of education, and the tutelary council; conversations with students involved in violent acts; longer classroom activities; posters; role-playing recreational activities to improve socialization and strict compliance with rules; enforcement of warnings; and expulsion and/or transfer to another school. These measures resulted in lower rates of violence in the investigated schools.^^35^^

Religious institutions are believed to have an important role in society regarding the discussion and reduction of violence in any social sphere. In 2018, the Fraternity Campaign, whose theme was Fraternity and the Overcoming of Violence, was promoted as a strategy to reduce the number of violent acts and discuss the different types of violence in Brazil.^^36^^

Occupational violence must be prevented and, for this purpose, governments that care about workers’ quality of life should be elected.^^37^^ It should be noted that, in this analysis, violence at work was considered to be any action, incident or behavior based on an instinctive attitude of the aggressor, as a result of which a professional is attacked, threatened, suffers some damage or injury during the development of his work. Still, it can be considered the result of the relationship of several factors, with emphasis on working conditions and the relationship between the worker and the aggressor.^^38^-^40^^

Victims of occupational violence who do not report the incident may experience decreased work-related self-confidence.^^41^^ Likewise, teachers exposed to physical and moral violence often do not report what happened or do not seek their rights for fear of reprisals from students or their families. Gurgel & Matos^^42^^ stated that, to prevent acts of violence, children should receive an education, especially in early childhood, that contributes to their process of socialization, making them more patient, as well as to the development of principles of morality and civility, which can effectively contribute to reducing aggressive behavior.^^43^^

Some researchers have been concerned with understanding the process of preventing workplace accidents and occupational safety. Brazilian companies must implement strategies to deal with such events, such as the ones established by current legislation, aimed at intervention and health monitoring at the organizational level.^^44^^ Structural changes that allow the development of more democratic and effective forms of social organization in the defense of fundamental rights to work and health are imperative, as well as the availability of balanced environments to act on and prevent occupational violence. In this sense, Brazilian schools and authorities must be allowed to exercise their responsibilities, which include granting, releasing, conceding, and inspecting. Therefore, policies based on occupational safety that promote democratic participation, information management, disarmament, equal human rights, and conflict resolution without using violence may reduce and prevent violence against teachers, students, and employees.

Finally, it should be emphasized that dealing with school violence is complex, since there are major social problems in Brazil that contribute to this phenomenon. All those involved should jointly develop plans to find solutions, and public policies addressing people’s real concerns and aimed at reducing school violence should be implemented. Common goals should be pursued to free schools from violence of any kind, whether physical, verbal, psychological, or sexual, among others.

### STRATEGIES TO PREVENT AND COMBAT BURNOUT SYNDROME

Burnout has numerous consequences on workers’ health, including musculoskeletal pain, insomnia, prolonged fatigue, and depressive symptoms,^^45^^ which can result in job dissatisfaction and absenteeism and make workers stop practicing their profession. Thus, qualified professionals within the school context can provide healthy coping strategies.^^46^^

For BS prevention strategies to be successful, BS should first be acknowledged as a reflection of the work environment and its specific causes need to be identified before solutions are proposed.^^47^^ The first step is identifying which stressors are present in the workplace and understanding the risks they pose to workers’ health.^^48^^ Secondly, the way workers respond to certain stressful situations should also be observed to understand their personal characteristics and emotional reactions.^^49^^ Planning and implementing actions based on these steps can help reduce or eliminate occupational stressors.^^48^^

Ribeiro et al.^^50^^ emphasize that companies should invest in projects aimed at maximizing quality of life and safety control, as well as professional growth and improvement within the company, which are essential for the development and success of any company. In this sense, it becomes clear that healthy, motivational, and adaptive programs aimed at resilience are necessary to deal with workplace stressors.

Providing a healthy environment for the development of occupational activities is essential and a right of every worker. Therefore, people’s physical, mental, and social well-being should be preserved, especially their mental health, considering that it has been little discussed by workers due to prejudice and its perception as an invisible problem.^^51^^ Prevention in mental health should also investigate the dynamics of workers’ relationship with their external environment, respecting the psychological and mental health of teachers.^^52^^ In this way, investigating the risks BS poses to teachers will contribute to the planning of strategies to preserve workers’ health.

Dealing with BS may include worker-focused intervention strategies, based on behavioral and cognitive coping skills, health education, physical activity, and meditation. Other tools are related to the relationship between worker, company, and teamwork; thus, plans should be prepared according to each individual need.^^48^^ However, it should be noted that the violence causing BS may not always be directly associated with the school environment, requiring investigations and public actions to act on the real source of violence.

Adopting healthy lifestyle habits regarding food, physical exercise, sleep, and leisure is also a measure to prevent the onset of BS, as they provide a break from the work routine. Another potential strategy is the promotion of meetings and events for teachers with a focus on how to recognize symptoms, where they can share their experiences with the group, which in turn may provide mutual support and solidarity, consequently reducing tensions in the workplace.^^48^^

Finally, to promote a better understanding of the programs that are often used to reduce or prevent BS, programs focused on the worker, the organization, or both are described.^^53^-^55^^ Organization-focused programs should seek changes in the organizational environment with the goal of reducing labor demand and increasing control of the level of participation in decision-making, such as work procedures, restructuring of activities, work evaluation, and supervision.^^53^^ These programs should be able to expand and qualify workers’ unique skills and coping strategies, expand social and emotional support, increase professional competence through continuing education, encourage activities outside of work (such as physical exercise, sports, and relaxation or hobbies), allow rest breaks, and determine goals.^^56^-^58^^

We believe this study has provided greater understanding of occupational violence among teachers and its influence on the development of BS. Strategies aimed at reducing violence should be planned and, to avoid the trivialization of violence in teachers’ routine, occupational violence should not be culturally accepted or seen as lacking solution. Therefore, we believe this study may be fundamental in the reduction of violent acts and, consequently, in the prevention of BS.

## FINAL CONSIDERATIONS

This study was able to identify which characteristics are related to the development of BS in teachers exposed to occupational violence, such as age, time of experience, employment relationship, schooling, type of school, class size, and variables of gender and race/skin color, sexual orientation, and religion or beliefs.

Occupational violence has been affecting teachers and may influence the development of BS. Thus, planning actions that involve teachers, students and their parents/legal guardians, employees, and managers is essential to promote a safe and healthier work environment. Government authorities should also act within their competences to ensure that violence is not seen as something natural or trivial, but rather as a phenomenon that impairs teaching and learning quality and causes illness and health problems in teachers and other workers.
